# Educational differences in mental health-related quality of life during the COVID-19 pandemic in Germany: the mediating role of pandemic-induced psychosocial stress

**DOI:** 10.3389/fpubh.2025.1535354

**Published:** 2025-05-01

**Authors:** Christina Kersjes, Ibrahim Demirer, Timo-Kolja Pförtner, Florian Beese, Jens Hoebel, Susanne Schnitzer, Elvira Mauz

**Affiliations:** ^1^Department of Epidemiology and Health Monitoring, Robert Koch Institute, Berlin, Germany; ^2^“Institute of Medical Sociology and Rehabilitation Science, Charité - Universitätsmedizin Berlin, Berlin, Germany; ^3^Center for Health Communication and Health Services Research, Department for Psychosomatic Medicine and Psychotherapy, University Hospital Bonn, Medical Faculty, University of Bonn, Bonn, Germany; ^4^Division of Research Methods, Faculty of Human Sciences, University of Cologne, Cologne, Germany

**Keywords:** quality of life, psychosocial stress, inequality, causal mediation analysis, COVID-19 pandemic, Germany

## Abstract

**Objectives:**

A large body of evidence shows poorer mental health among lower socioeconomic groups, with chronic stress being an important pathway in this relationship. It was expected that the mental health of people with low socioeconomic status may have been particularly affected by the COVID-19 pandemic. While it has been established that stress also impacted mental health during the pandemic, the aim of this study was to analyze if pandemic-induced psychosocial stress (PIPS) mediated educational differences in mental health-related quality of life (MHRQOL) and which life domains were particularly affected.

**Methods:**

The data came from the population-based representative study “Corona Monitoring Nationwide – Wave 2 (RKI-SOEP-2),” from November 2021–February 2022, restricted to the working age population (18–67 years, *n* = 7,425). The mediating role of PIPS in educational differences (fractional rank variable from 0 [lowest] to 1 [highest education]) in MHRQOL was assessed for the life domains family, partnership, financial situation, work/school, social life, and leisure time. We used causal mediation analysis to estimate the total, indirect, and direct effects.

**Results:**

MHRQOL increased with higher education. Higher education was associated with more PIPS in the domain’s family, social life, and leisure time, while lower education was associated with more financial PIPS. PIPS in the life domains family, financial situation, leisure time, and social life significantly mediated educational differences in MHRQOL. No significant mediation effect was found for partnership and work/school PIPS.

**Conclusion:**

PIPS contributed to educational differences in MHRQOL, with different educational levels showing more stress in different life domains. Group-targeted and life domain-specific approaches for prevention and intervention should be considered.

## 1 Introduction

Health inequalities to the detriment of socially disadvantaged groups are empirically well documented, including for mental health [e.g., (1)]. This also applies to Germany, where persons with low socioeconomic status (SES) indicated by education, income, or occupation have a significantly higher prevalence of any mental disorder (2). Persons with low education have a 2.5 times higher prevalence of depressive symptoms (3). Differences by SES are also detected in health-related quality of life (HRQOL) (4–6), a multidimensional construct that often includes physical, psychological, and social factors (7) and that reflects the burden of disease (8).

The mechanisms underlying mental health inequalities are manifold. Beyond structural (e.g., societal and geographical) circumstances, factors at the individual level are chronic stress and maladaptive coping mechanisms (9). Disadvantaged populations like persons of low SES are more likely to experience adverse environmental conditions (e.g., insecure neighborhoods, precarious work, limited access to green spaces and education) (10). This may lead to chronic activation of the stress response system, contributing to allostatic load, which impacts various physiological regulatory mechanisms. Low education has, for example, been linked to higher biological risk in the endocrine, inflammatory, cardiovascular, and metabolic systems, which are all linked to allostatic load (11). This, in turn, increases susceptibility to mental and neurodegenerative disorders (9). Additional to facing more environmental challenges, people of low SES often report less psychosocial resources, such as resilience or coping abilities (12–14). For example, low SES has been linked to maladaptive coping abilities like ruminative or avoidant coping (12, 13). Resilience, in turn, has been identified as a mediator of socioeconomic differences in HRQOL (14).

When the COVID-19 pandemic hit the world in March 2020, the pandemic itself, as well as the non-pharmaceutical interventions implemented by governments to contain infections, had a huge impact on people’s lives and their physical and mental health (15). The non-pharmaceutical interventions in Germany and often worldwide included physical distancing or lockdowns, closures of nurseries and schools, remote work, and shutdowns of shops, services, restaurants, as well as leisure-time facilities (15, 16), affecting a wide range of life domains. Given this added number of stressors, a rise in mental health problems was expected (17). It was furthermore feared that this rise would particularly hit persons with low SES (18), since they face higher risks for severe morbidity and mortality from COVID-19 due to their higher burden of pre-existing health conditions (19, 20), as well as the financial ramifications of the pandemic (21, 22).

However, a clear exacerbation of socioeconomic inequalities in mental health was not found consistently during the pandemic, with empirical findings showing mixed results (23). While some studies did find differences by SES in mental health changes during the pandemic to the detriment of those with a lower status (24, 25), others found no differences (26, 27) or detriments to people with higher SES (28–31). In Germany, a persistent social gradient to the detriment of persons with low education per depression and anxiety was found during the pandemic (32). However, an early and continuous increase in depressive symptoms among the highest education group stood out. The studies varied in SES indicator, outcome, the pandemic wave observed, and country-specific context. A few studies addressed HRQOL and its risk factors. Regarding high-income countries, a deterioration of HRQOL in the general population during the COVID-19 pandemic compared to pre-pandemic norms was found in Denmark (33), Portugal (34), Canada (35), Japan (36), and in cross-country comparisons, including Germany (37, 38). In all studies, the HRQOL dimension concerning mental health was particularly affected. Risk factors were, among others, lower SES (36), job loss, working from home, change in work situation (33), and income difficulties (37). Results were particularly unclear regarding education. While Ferreira et al. (34) found low education to be a risk factor, Violato et al. (38) found females with high education to experience worse HRQOL. König et al. (37) did not find education to be a significant determinant for mental health-related quality of life (MHRQOL).

Identifying the mechanisms underlying SES differences in mental health may help to shed light on these heterogeneous findings. Also, during the pandemic, it was established that psychosocial stress impacted mental health (39, 40), and that there were differences in pandemic-induced psychosocial stress (PIPS) according to SES (41). However, to our knowledge, it was not observed whether PIPS mediated socioeconomic differences in MHRQOL and whether this varied by different life domains, such as leisure time, work, family life, or one’s financial situation. The present study therefore sought to examine, first, if there were educational differences in MHRQOL during the COVID-19 pandemic in Germany. Second, we examined if pandemic-induced stress in the life domains family, partnership, financial situation, work/school, social life, and leisure time mediated the relationship between education and MHRQOL. Different indicators of SES represent both the general ranking within society as well as specific socioeconomic conditions determined by the nature of the measure (42). For example, income rather reflects material, education immaterial aspects of SES. Using a construct comprising multiple indicators may complicate interpreting specific effects. We explicitly focused on education as a key dimension of SES, since education is at the beginning of the causal chain of people’s status attainment and its pathway to health (e.g., educational attainment influences people’s occupation and income) (43, 44). Moreover, it is a more stable indicator than income and occupation, that may have fluctuated particularly during the pandemic. We expected pandemic-related changes in work or income to be partly reflected in the mediator PIPS (e.g., domain financial situation and work/school).

## 2 Materials and methods

### 2.1 Study design

The data came from the study “Corona Monitoring Nationwide – Wave 2 (RKI-SOEP-2),” a population-based study conducted by the Robert Koch Institute, the German Institute for Economic Research, the Institute for Employment Research, and the Research Center of the Federal Office for Migration and Refugees. Participants were a subsample of the German Socio-Economic Panel (GSOEP) at the German Institute for Economic Research, an annual representative cohort of private households in Germany since 1984 (45). All GSOEP households in the gross sample were invited to participate in the RKI-SOEP-2 study.

Data collection took place between November 2021 and February 2022. Besides biospecimen (dry blood sample), collection included data from self-administered questionnaires either in paper-and-pencil or online format. Topics were, among others, SARS-CoV-2 infections, COVID-19 vaccination or willingness to vaccinate, health issues, (health) behaviors, and burdens during the pandemic [for details, see (46)]. We restricted the sample to (1) the working age population of 18–67 years under the assumption that this population was particularly affected by pandemic-specific stressors like changes in work (e.g., home office, short-term work), financial instability, and closures of childcare facilities, and (2) participants with complete data.

### 2.2 Variables

#### 2.2.1 Outcome: mental health-related quality of life

To measure MHRQOL, we used the German version (47) of the mental component summary scale of the 12-item Short Form Health Survey (SF-12) (48). The scale is composed of the four subscales vitality (one item), social functioning (one item), role limitations because of emotional problems (two items), and mental health (two items), which were combined and transformed. Values were standardized to a representative population of the year 2004, and ranged from 0 to 100, with a mean of 50 and a standard deviation of 10 (47), with higher values indicating a better MHRQOL. For details of the procedure, see Nübling and Mühlbacher (47). It is a validated and internationally widely used instrument (Cronbach’s alpha = 0.78) (49).

#### 2.2.2 Exposure: education

Educational attainment was measured with the 2011 version of the International Standard Classification of Education (ISCED) (50). The participants’ highest educational qualification was categorized as low (lower secondary education or below, ISCED levels 0–2), medium (upper secondary or post-secondary education, ISCED levels 3–4), or high (tertiary education, ISCED levels 5–8) education (51). This variable was used to display sample characteristics. To facilitate the interpretation of the analysis, we converted the variable to a continuous fractional rank variable with ridit analysis (52), ranging from 0 to 1, with higher scores indicating higher educational attainment. Ridits for a category were calculated as the proportion of individuals in a lower category plus half of the individuals in the category itself, divided by the grand total. They therefore represent the midpoint of the cumulative proportion of an education category relative to the entire distribution of the variable. Ridit Scores were 0.812 for high, 0.362 for medium and 0.050 for low education. The ridit score for the medium education group, for example, means that, on average, 36,2% have a similar or lower education. The ridit-transformed education variable was used for all further calculations (correlation, regression, mediation and sensitivity analysis).

#### 2.2.3 Mediator: pandemic-induced psychosocial stress

PIPS was measured with one survey question: “Overall, how much have you been stressed due to the pandemic, with regard to … family, partnership, financial situation, work/school, social life, psychological well-being, and leisure time activities?” The rating scale for each life domain ranged from “not stressed,” “a little stressed,” “rather stressed,” to “strongly stressed” and included “not applicable” as a response option. We handled the answer “not applicable” as “not stressed” instead of treating it as missing. The latter would have caused a systematic exclusion of persons without a job or partnership, since those persons most likely would choose “not applicable” for those domains. We did not include the life domain “psychological well-being” in the analysis due to construct overlap with the outcome.

#### 2.2.4 Covariates

To control for exposure-outcome, mediator-outcome, and exposure-mediator confounding (53), we included the following set of covariates: age in years, sex (male, female), and migration background (direct migration background: people born in a different country than Germany; indirect migration background: people born in Germany with one or both parents with direct migration background; no migration background: people born in Germany with no migration background of the parents). In a sensitivity analysis, we also included pre-pandemic MHRQOL by linking the data intra-individually to panel data from the GSOEP core wave 2018, the last assessment of MHRQOL before the pandemic.

### 2.3 Statistical analysis

For all analyses we used weighting factors to compensate for systematic non-response at individual and household levels and to adjust the sample to match the German micro-census according to age, sex, migration status, federal state, household type and size, and owner-occupant housing (54). Standard errors were estimated that accounted for weighting and household-clustering. We first calculated correlations between education, PIPS and MHRQOL. To assess educational differences in MHRQOL, we fitted bivariate and multiple linear regressions. We then applied causal mediation analysis. Causal mediation analysis allows one to identify and evaluate assumptions to establish causality of a mediator and provides parametric and non-parametric estimation methods, as well as a sensitivity analysis (55). We first fitted linear regression models separately for each PIPS domain to analyze: (1) the association of education and PIPS with adjustment of covariates and (2) the association of education and MHRQOL during the pandemic, including the respective PIPS domains as mediators and covariates and exposure-mediator interaction. We then performed causal mediation analysis of each life domain based on a counterfactual framework (56). This allows one to obtain the direct [average direct effect (ADE)] and indirect effect [average causal mediation effect (ACME)], as well as the total effect (sum of ADE and ACME) (56). To test the statistical significance of these effects, we used a non-parametric bootstrap procedure with 1,000 repetitions. We tested for multicollinearity using variance inflation factor. Since a categorical variable was included, we calculated the adjusted generalized variance inflation factor (GVIF^(1/(2*Df))^) for each applied model, but excluding interaction terms (57).

We conducted two sensitivity analyses, including:

1. A sensitivity analysis regarding the results of the mediation analysis (a) against unobserved pre-exposure confounders affecting mediators and outcome (sequential ignorability), (b) significant exposure-mediator (XM) interaction, and (c) against exposure-induced mediator-outcome confounding (53). (a) Was based on the correlation of the error term of the mediation and outcome model, called rho (*ρ*). The smaller the rho, the more sensitive was the study to unobserved confounding (56), with other studies finding 0.3 to be “a fairly robust estimate” (58) or a “modest violation” (59); (b) XM interaction tests for differences in the mediated effects that depend on the combination of the mediator and exposure level (60), for instance, if the mediation effect for lower education and high stress is different than for higher education and high stress; to test the robustness of the results against (c) exposure-induced mediator-outcome confounding, we composed a sub-sample of ours with persons with data on pre-pandemic MHRQOL and included it as a covariate in the mediation analysis.
2. The second sensitivity analysis concerned sample selectivity. For this purpose, we compared the original RKI-SOEP-2 sample against the final sample. For continuous variables, we calculated experimental selectivity based on Lindenberger et al. (61), that expresses the part of the selectivity that is not due to selection to the study (selection bias) or panel attrition but due to having consistent information on the analysis variables. For this purpose, we subtracted the mean of the variable in question of the original sample (S_1_) from the mean of the final sample (S_2_) and divided it by the standard deviation of the original sample. The magnitude of the experimental selectivity effect was standardized according to Cohens d, with |d| = 0.2 being a small, 0.5 a medium, and 0.8 a large effect size (62). For categorical variables, we used absolute population differences as a descriptive measure.

We conducted all analyses in R (Version 4.3.0) (63). For causal mediation analysis, we used the mediation package in R (Version 4.5.0) (64).

## 3 Results

### 3.1 Descriptive statistics

**Figure 1:**
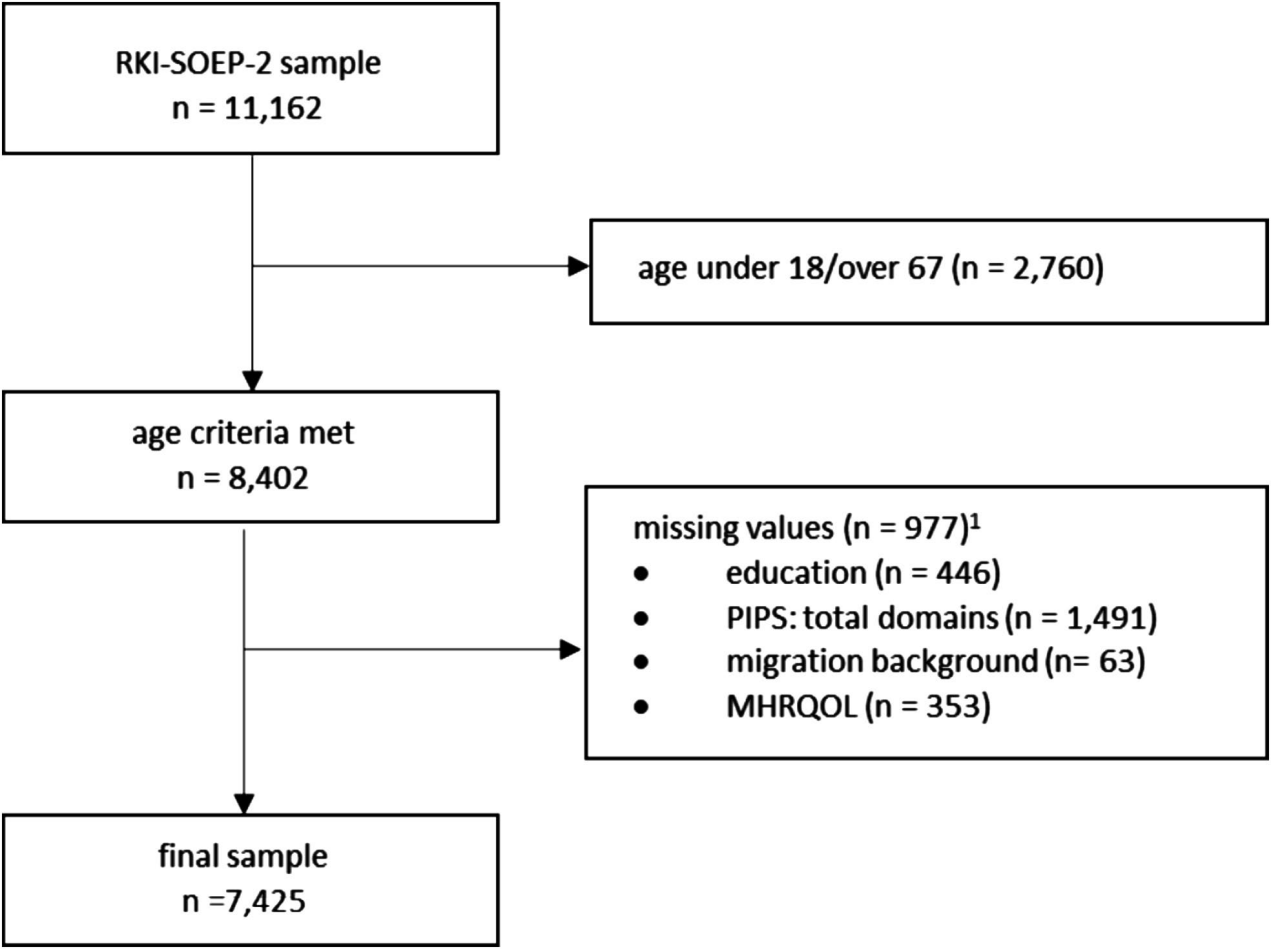
Flow chart of the sample selection. ^1^ The values do not add up to 977 due to multiple missing data points from one person.

The final sample consisted of 7,425 participants (see Figure 1).

Sample characteristics can be found in Table 1. The highest levels of PIPS were found in the life domains leisure time (M = 3.039, SD = 0.924) and social life (M = 3.004, SD = 0.858). PIPS and MHRQOL for the different sexes, ages, and educational groups can be found in Appendix Table A1. Correlations between education, PIPS and MHRQOL can be found in Appendix Table A2. Adjusted generalized variance inflation factors (see Appendix Table A3) indicated no multicollinearity, since they did not exceed the conservative threshold of 1.6 (57).

**Table 1:** Descriptive statistics of the study population (*n* = 7,425).

Characteristics		*N* (%)^1^ or mean (SD)^2^
Sex	Male	3,327 (50.5%)^1^
	Female	4,098 (49.5%)^1^
Age groups	18–29 years	1,060 (18.7%)^1^
	30–39 years	1,110 (20.6%)^1^
	40–49 years	1,643 (19.2%)^1^
	50–67 years	3,612 (41.5%)^1^
Migration background	No migration background	6,191 (76.3%)^1^
	Direct migration background	791 (16.8%)^1^
	Indirect migration background	443 (6.9%)^1^
Education	High (ridit score: 0.812)	3,096 (37.6%)^1^
	Medium (ridit score: 0.362)	3,657 (52.4%)^1^
	Low (ridit score: 0.050)	672 (10.0%)^1^
PIPS	Family	2.621 (0.904)^2^
	Partnership	1.914 (0.961)^2^
	Financial situation	1.883 (0.962)^2^
	Work/school	2.297 (1.126)^2^
	Social life	3.004 (0.858)^2^
	Leisure time	3.039 (0.924)^2^
MHRQOL	2021/2022	46.177 (10.817)^2^

^1^*N* (%): *N* = unweighted *N*, % = weighted %; 2mean (standard deviation (SD)); % figures do not necessarily add up to 100% due to rounded values.

### 3.2 Educational differences in MHRQOL

Compared to high education (M = 46.857, SD *=* 10.285), persons with medium (M = 46.049, SD = 10.906) or low education (M = 44.294, SD = 12.005) had a lower MHRQOL during the pandemic. Results of the regression analysis with the continuous education variable can be found in Table 2. A significant association of higher education with higher MHRQOL was found both in the bivariate and the multiple regression model with controls for sex, age, and migration background.

**Table 2:** Results of the regression analysis of MHRQOL on education.

Variable		Bivariate model	Multiple model
		Beta (SE)	Beta (SE)
Constant		44.877*** (0.431)	40.314*** (0.779)
Education		2.601*** (0.727)	1.539* (0.708)
Sex	Male		Ref.
	Female		−2.857*** (0.350)
Age			0.149*** (0.014)
Migration background	No		Ref.
	Direct		−0.617 (0.562)
	Indirect		0.170 (0.809)

SE = standard error; Ref. = reference category; *p < .05, **p < .01, ***p < .001. Significant coefficients (considering a .05 significance level) appear in bold. Multiple regression model adjusted for sex, age, and migration background.

### 3.3 Mediation analysis

**Figure 2:**
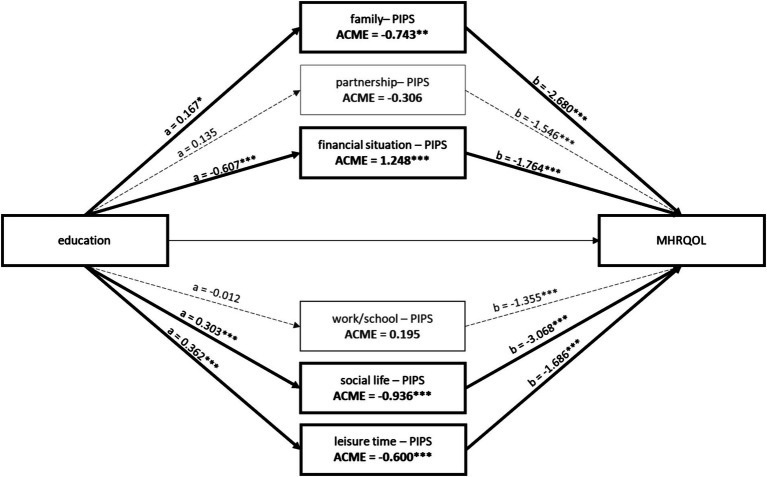
Coefficients of the a-paths and b-paths, as well as ACMEs of the mediation analysis. ACME, average causal mediation effect. Nonsignificant paths and ACMEs are depicted in gray and in dashed lines. Results adjusted for sex, age, and migration background.

Figure 2 displays the coefficients between education and each PIPS domain (a-path) and between each mediator and MHRQOL (b-path). Regarding the a-path, there were differential effects of education on PIPS. Education was negatively correlated with PIPS in the domain’s financial situation, indicating the higher the education, the less stress individuals experienced. Education was positively correlated with PIPS in the domain’s family, social life, leisure time, and at a 10% significance level, partnership (*p* = 0.055), indicating that higher education was associated with more stress. Regarding the b-path, all coefficients between PIPS and MHRQOL were negative and significant, thus PIPS in all life domains was negatively associated with MHRQOL.

Table 3 summarizes the results of the mediation analyses for each of the six life domains. PIPS in the life domains family, financial situation, social life, and leisure time significantly mediated the relationship between education and MHRQOL, although in different directions. PIPS in the life domain partnership was only significant at the 10% level (*p* = 0.092). There was no significant mediation effect for stress regarding work/school. The negative ACME (e.g., in the domains family, social life, and leisure time) led to a reduction of educational inequalities in MHRQOL, while the positive ACME (e.g., in the domain financial situation) led to an increase. Considering the indirect effect of education on MHRQOL, the direct effect was positive and significant for all life domains except financial situation. All total effects (direct + indirect effects) were positive and significant.

**Table 3:** Results of the mediation analysis of educational differences in MHRQOL due to PIPS in different life domains.

PIPS life domain	Effect type	Beta	95% CI	*p*-value
Family	ACME	**−0.743**	[−0.878; −0.090]	<0.01
	ADE	**2.349**	[0.520; 3.410]	<0.01
	Total effect	**1.606**	[0.040; 3.030]	<0.05
Partnership	ACME	−0.306	[−0.614; 0.050]	0.092
	ADE	**1.971**	[0.483; 3.320]	<0.01
	Total effect	**1.665**	[0.130; 2.960]	<0.05
Financial situation	ACME	**1.248**	[0.935; 1.750]	<0.001
	ADE	0.502	[−1.246; 1.630]	0.776
	Total effect	**1.749**	[0.100; 2.930]	<0.05
Work/school	ACME	0.195	[−0.210; 0.230]	0.928
	ADE	**1.449**	[0.071; 2.970]	<0.05
	Total effect	**1.644**	[0.106; 2.920]	<0.05
Social life	ACME	**−0.936**	[−1.220; −0.490]	<0.001
	ADE	**2.445**	[−0.965; 3.740]	<0.001
	Total effect	**1.509**	[0.089; 2.930]	<0.05
Leisure time	ACME	**−0.600**	[−0.941; −0.380]	<0.001
	ADE	**2.187**	[0.719; 3.560]	<0.01
	Total effect	**1.588**	[0.102; 2.920]	<0.05

CI = confidence interval; ACME = average causal mediation effect; ADE = average direct effect. Significant coefficients (considering a .05 significance level) appear in bold. Results adjusted for sex, age, and migration background.

### 3.4 Sensitivity analysis

1. We tested the sensitivity of the mediation analysis results via (a) rho, (b) XM interaction, and (c) exposure-induced mediator-outcome confounding. (a) The estimates for rho can be found in Table 4. We found a slight indication of unobserved confounding between mediator and outcome model. Rho mainly amounted to −0.2, with four estimates being −0.1; (b) all XM interactions were nonsignificant (see Appendix Table A4). Thus, an equal mediation between different levels of education and the PIPS domains can be assumed. (c) Results of the mediation analysis after including pre-pandemic MHRQOL as an exposure-induced mediator-outcome confounder can be found in Appendix Table A5. Results did not differ in terms of content from those of the main analysis.
2. To evaluate sample selectivity, we assessed experimental selectivity (see Appendix Table A6). The effect sizes regarding MHRQOL and all PIPS domains varied between |d| = 0.033 and |d| = 0.126 and were below an even small effect size, indicating no selectivity between the original sample (S_1_) and final sample (S_2_). Regarding population differences, a slightly higher selectivity in persons with low education (N_S1_ = 967 vs. N_S2_ = 672) and persons aged 18–29 years (N_S1_ = 1,319 vs. N_S2_ = 1,060) stood out.

**Table 4:** Results of the sensitivity analysis to the violation of the non-parametric sequential ignorability assumption via rho.

PIPS life domain	Rho	
	Control	Treated
Family	−0.2	−0.3
Partnership	−0.1	−0.3
Financial situation	−0.2	−0.2
Work/school	−0.1	−0.1
Social life	−0.2	−0.2
Leisure time	−0.1	−0.2

The sensitivity analysis included weighting factors but not household clustering. Results adjusted for sex, age, and migration background.

## 4 Discussion

### 4.1 Summary of results

We found significant educational differences in MHRQOL during the pandemic, with persons with higher education experiencing better MHRQOL. Educational differences were partly mediated by PIPS per the life domains family, financial situation, social life, and leisure time. The mediation effect of partnership stress was only significant at a 10% level. Work/school stress did not mediate educational differences in MHRQOL. The mediating effects differed according to life domain. Higher education was associated with higher levels of stress in the domain’s family, social life, leisure time, and partnership (at a 10% level of statistical significance) and lower levels of stress in the domain financial situation. Since we did not find significant interactions, the mediation effect itself was similar for all educational levels. The results are in line with other studies which found educational differences in symptoms of depression (32), anxiety and depression (25), and HRQOL (34) during the pandemic. It details the mechanisms by which these educational differences may have occurred in the first pandemic phases.

### 4.2 PIPS per different life domains

#### 4.2.1 PIPS regarding social life

Physical distancing measures had a significant impact on social life (e.g., socializing with family, friends, and colleagues), contributing to an increase in social isolation and loneliness (65). A lack of social connectedness is well proven to have negative effects on health outcomes such as sleep, cognition, increased stress, depression, anxiety, and suicidal ideation and behavior (66–68). In Germany, a rapid increase in loneliness was observed during the pandemic. The percentage of people feeling at least sometimes lonely rose from 14.2% in 2017 to 40.1% in March 2020, and the levels persisted during the pandemic (69). While before the pandemic a social gradient existed with persons with low education being the loneliest and persons with high education the least lonely, assimilation appeared to occur during the pandemic, leading to a decrease of this socioeconomic gradient, both by education and income (69). Our findings align with those of Entringer. They are also in line with the greater increase in symptoms of depression among persons with high education in Germany until mid-2022 (32). Possible reasons for this phenomenon may include the transition to home office work and a reduction in social participation for persons with high education. People with high education were more likely to be able to work from home (70, 71). This may have contributed to a lack of interaction with colleagues or clients. Persons with low education were less able to work in a home office (71), thus they possibly maintained more work-related contacts, contacts outside the home, and other weak social ties, which have been proven beneficial to well-being (72, 73). Furthermore, since restaurants, bars, cultural institutions, etcetera, were closed during lockdowns, eventual advantages of persons with high education regarding social participation may have resolved (69). Our results support that the physical distancing measures may have led to undesired side effects like mental distress in social life and loneliness, affecting mental health with a presumably greater burden on people with higher education. This is particularly important since loneliness seemed to persist (74) and contribute to depression (75) even after the lifting of lockdown measures.

#### 4.2.2 PIPS regarding leisure time

Social distancing measures also affected leisure time activities. Before the pandemic, it was shown that persons with higher education engaged more in leisure time physical activity (76) and recreational activities like sports, dancing, theatre, community activities, and volunteer work (77). Participation in such activities has been linked to a better quality of life (77). The restrictions during the pandemic may have caused a greater loss in these resources for persons with high education—another explanation for a more marked deterioration in mental health in the highly educated group (32, 69). This is consistent with studies that drew on the conservation of resources theory in order to explain a greater increase in depression among people with high education during the pandemic (31). According to this theory, well-being decreases when individual resources are threatened or lost, and its effect may depend on the extent of resource loss (78, 79).

#### 4.2.3 PIPS regarding family and partnership

We found a significant mediation effect of PIPS regarding family and at a 10% level of significance for partnership. Persons with higher education showed higher stress levels in both domains (a-path). The latter results differ from those of Beese et al. (41) who found prevalence ratios to the detriment of persons with low education. These opposing results can be explained by a differing operationalization of PIPS. Beese et al. compared highly stressed persons with those who were not, slightly stressed, and rather stressed. However, the highly educated people seemed to be especially slightly and rather stressed in both domains, which can be derived from the supplementary analyses of Beese et al., where none/slight family PIPS was compared to rather/high family PIPS. The association tended to shift towards a positive correlation between education and family PIPS and a lower prevalence ratio for partnership stress.

Before the pandemic, empirical findings showed a social gradient in family and partnership stress: low-SES families as opposed to high-SES families had disadvantages regarding parenting practices, family conflict, and day-to-day routines (80, 81). Higher educational attainment was associated with greater stability and satisfaction and less educational attainment with more problems, instability, and less happiness in marriage or other romantic relationships (82). During the pandemic, however, people with a higher educational level seemed to be particularly affected. Perhaps the pandemic-related differences in access to childcare in daycare centers depended on the respondent’s occupation. Access to emergency childcare was found to positively affect parental well-being during the pandemic (83). Emergency childcare was only available to employees in critical infrastructure, such as hospitals, supermarkets, and the transport sector. Holst et al. (84) found that emergency childcare was most often used by persons who worked in non-academic service occupations. People with higher educational attainment were more often able to work from home (71) and therefore had little access to emergency childcare. Thus, juggling childcare and personal job demands was a particular challenge that would not have existed to the same extent under other circumstances. Accordingly, Schwarz et al. (85) found higher work-to-family conflicts for parents with a high occupational status or who were teleworking during the pandemic. In addition, according to the family investment model, persons with high SES (e.g., greater education) try to promote their children’s academic, social, and career success more (86). At this point, various factors may have increased family stress. For example, increased efforts may have been made to maintain this standard of support through parental support, which in turn may have increased parents’ stress levels. Otherwise, not being able to maintain this standard during the COVID-19 pandemic without access to the usual support facilities, such as extra tuition, art, or music schools, sports courses, etcetera, could in itself have been experienced as stressful.

Family stress may also have influenced partnership stress (87). We found that the correlation between the two PIPS domains was among the highest, indicating that the possible mechanisms might have influenced each other. A further reason affecting partnership quality might be an unequal division of housework and childcare, which particularly developed in previously more egalitarian partnerships to the detriment of women’s contribution (88), thus possibly contributing to an overall increased partnership stress in the high education group.

#### 4.2.4 PIPS regarding financial situation

Persons with lower education reported higher PIPS levels regarding their financial situation. This is in line with international findings. A higher likelihood to experience financial threats (e.g., working in sectors that were shut down during the pandemic, such as restaurants, hotels, retailers, and arts and leisure services and with fewer options to work from a home office) (89) was found for persons with lower education (90), income (91) and occupational prestige (22). Additionally, these individuals may have been hit harder due to having less of a financial buffer and less leeway to cut down costs (91). Several studies have shown that financial stress or worries are a significant mediator between financial threats (e.g., unemployment rate, individual job loss or loss of income, living at risk of poverty) and mental health (72, 91–93), which is supported by our results. Also, for Germany, pandemic-induced job and income insecurity were found to decrease mental health (37, 94). Wörn et al. (90) found similar results to ours: Persons with low education had a higher risk of financial threats (a 2–3 times higher risk of losing their job), but once a job loss occurred, its negative effect on mental health was similar across educational levels.

#### 4.2.5 PIPS regarding work or school

The mediation effect for the domain work/school was nonsignificant. This was surprising since many changes in workplaces occurred during the pandemic. The relationship between work/school PIPS and MHRQOL was significant, with more stress indicating a worse MHRQOL. However, there were no significant educational differences in PIPS, rendering the indirect effect nonsignificant. It is possible that both educational and occupational groups experienced similar levels but different kinds of stressors during the pandemic. While fear of infection and workplace insecurities were more of a concern for non-academic workers (84, 89), so was working from home for persons with high education. The latter has been connected to poor mental health (94, 95). Perhaps it was not so much the changes in the work situation itself that lead to PIPS, but associated worries may have resulted in an increase in PIPS in other life domains. Lack of social interaction at work and difficulties in reconciling work with household and family chores when working from home (96) may be reflected in the life domains social life and family. Financial worries associated with work changes may be reflected in the life domain financial situation. The latter is supported by findings from Dragano et al. (94) who found short-term work only to be associated with poorer mental health when no short-term allowance was paid.

### 4.3 Implications

Overall, our results show that pandemic-related stress and its mediating effect manifested itself in varying directions and degrees in different areas of life among educational levels [also see (41)]. Therefore, our results support the need for target-group-specific prevention and intervention measures per different life domains. The main focus in the high education group is more likely to be seen in the family and social contexts. Education used to be a protective factor before the pandemic, and it still acted as one during the pandemic, as our positive direct effects of education on MHRQOL indicate. However, a loss of resources seems to have occurred, particularly in the domain’s social life and leisure time. It is still unclear what the medium- to long-term effects of these losses are, especially regarding loneliness (69). Besides continuous monitoring (69), it is important to enhance interventions that support social connectedness despite physical distancing (97) and offer sufficient mental health support and services in communities. Interventions that proved successful to reduce loneliness included psychological therapy interventions (e.g., mindfulness, Tai Chi Qigong, laughter therapy), social facilitation software (e.g., videoconferencing), educational programs (e.g., lessons on friendship), robotic pets, and gardening (98). Particular caution should be taken when closing childcare facilities and schools. If these are absolutely necessary for reasons of infection control, they should be accompanied by measures that alleviate the added burden on families. These may range from economic support, access to emergency daycare, enabling reduced working hours and home office work, and family counseling (99), to providing parenting resources (e.g., guidance on conflict management, work-life balance) (100).

The main focus for people with low education and SES should be on aspects of their financial security. Although short-term compensation seems to have buffered negative effects (94, 101), persons with low education were still more stressed in their financial situation possibly due to a loss of income (since short-term compensation only partly covered the income loss), the increase in job insecurity, and actual job loss. Corresponding labor and social security measures may not only benefit a country’s economy but also reduce the impact of financial worries on mental health. People with low SES should have been especially targeted in the response to the COVID-19 pandemic and, above all, regarding their medium- and longer-term financial consequences. While the majority of burdens on the higher education groups have presumably eased again as the pandemic has subsided (e.g., childcare facilities have reopened, access to previously available resources has been restored), it cannot be ruled out that the financial burdens and concerns of the lower education group have continued to increase. The COVID-19 pandemic was accompanied by an economic recession (102). On top of that, the war in Ukraine has been accompanied by high inflation, causing price increases in energy and food (103). Epidemiological studies on the impact of the recession due to the global financial crisis of 2007/2008 showed a deterioration of mental health and rise in suicide rates for several years after, with vulnerable groups like the unemployed and persons with great financial insecurities (104). Hence, there is a risk that the MHRQOL gap may widen in the aftermath of the COVID-19 pandemic to the detriment of people with low education and SES. Continuous monitoring of these effects on mental health with a particular focus on vulnerable groups is therefore crucial.

### 4.4 Strengths and limitations

A strength of the study was its large representative sample with data from a more advanced stage of the pandemic, given that a lot of research on the COVID-19 pandemic focused on the earlier stages. While the temporal sequence between exposure and mediator was preserved, mediator and outcome were assessed at the same time. It was therefore impossible to determine causality, and the results are rather suggestive of the process. Although reverse-causality and reciprocity in the cross-section of the process between PIPS and MHRQOL during the COVID-19 pandemic cannot entirely be ruled out, the policy implications of the results remain clear. In a sensitivity analysis, we were also able to include the pre-pandemic mental health status variable, thus adjusting for the reciprocity between MHRQOL and PIPS. The results proved robust on an interpretation level.

Also noteworthy is that the survey question used in this study to assess PIPS was not a validated measurement instrument, which poses a threat to its validity. It is unclear how the question was interpreted in regard to reporting time period and content. Since the question did not refer to a specific time frame, people may have answered in reference to their most stressed time during the pandemic, on average, or with a primacy or recency bias. They may also not have been able to properly differentiate between pandemic-induced stress and general stress and/or between the meaning of the individual life domains (e.g., social life, leisure time activities). The interpretation of the answer category “not applicable” is also unclear. As described, we assigned this category to “not stressed” instead of treating it as missing to not systematically exclude people. If this assignment altered the results, it probably made the result estimation more conservative. Sensitivity analysis in another study (41) showed that the results did not differ significantly when assigning “not applicable” to “not stressed” or treating it as missing.

A sensitivity analysis also showed that our results were rather robust per most life domains. We only found a slight indication of unobserved confounding between mediator and outcome. Experimental selectivity assessment indicated no significant effect regarding MHRQOL and all PIPS domains; results can therefore be extended to the original sample. Besides experimental selectivity, selectivity may also have occurred due to a selective willingness of participants of the GSOEP core study to participate in the RKI-SOEP-2 study. To correct the data, extensive information at individual and household levels from the GSOEP core study was used to identify predictors of non-contact, panel attrition, and refusal to participate, and weights were calculated (54).

Due to the complexity of this study of six life domains, we did not stratify by gender and age. However, differences among those variables are to be expected. Future research should keep this in mind when investigating mechanisms. Gender-related effects can be especially expected in the dimension family stress and age-related effects per the dimensions social and leisure time. We furthermore recommend exploring the mechanisms more in detail within the life domains (e.g., regarding household composition, job loss, short-term work). Additionally, with education, we only used one indicator of SES as exposure variable, that unlikely provides a comprehensive description of the socioeconomic circumstances (42). Particularly aspects regarding material standard of living and working conditions may not have been sufficiently covered. Status inconsistencies such as high education but low income, are not reflected by just one indicator. Pathways from other SES indicators to MHRQOL may differ. Beese et al. (41), for example, showed that there were significant differences in PIPS according to income and that these may also differ compared to educational differences (e.g., groups with lower income experienced more social life PIPS). SES differences by income or occupation were beyond the scope of this paper. However, we assume that self-employed people or persons working in specific sectors may have been vulnerable to certain pandemic-related stressors. Future research should include different SES indicators and address these questions.

## 5 Conclusion

Our study helped to identify life domains that had an impact on mental health differences during the pandemic. We found educational differences in MHRQOL and were able to identify some underlying mechanisms. We found higher education to be associated with more stress in social life, leisure time, and family life, and lower education with one’s financial situation. These findings indicate that public health strategies in pandemic times, but also before (pandemic preparedness planning) and beyond, should consider the psychosocial mechanisms of mental health differences and provide support with group-targeted and life domain-specific measures.

## Data Availability

The datasets presented in this article are not readily available because informed consent from participants did not cover the public deposition of data. However, the data underlying the analysis in this article is archived in the SOEP Research Data Centre in Berlin. Requests to access the datasets should be directed to https://www.diw.de/en/diw_01.c.601584.en/data_access.html.
