# Supplementary material for: Educational differences in mental health-related quality of life during the COVID-19 pandemic in Germany: the mediating role of pandemic-induced psychosocial stress
Source: Front Public Health. 2025 May 1;13:1535354. doi: 10.3389/fpubh.2025.1535354 (PMC12078178; doi:10.3389/fpubh.2025.1535354)
Supplement: Supplementary file 1 [file Table_1.docx]

**Supplementary materials – Appendix**

**Table A1.** Means (and standard deviations) of PIPS and MHRQOL by sex, age, and education groups

| characteristics | | family | partner-ship | financial situation | work/  school | social life | leisure time | MHRQOL |
| --- | --- | --- | --- | --- | --- | --- | --- | --- |
| sex | male | 2.485 (0.866) | 1.907 (0.937) | 1.856 (0.963) | 2.240 (1.101) | 2.950 (0.861) | 2.955 (0.933) | 47.596 (10.277) |
|  | female | 2.759 (0.921) | 1.921 (0.985) | 1.911 (0.960) | 2.356 (1.148) | 3.059 (0.851) | 3.124 (0.907) | 44.728 (11.159) |
| age group (in years) | 18-29 | 2.437 (0.852) | 1.730 (0.939) | 1.814 (0.955) | 2.842 (1.070) | 3.172 (0.788) | 3.211 (0.865) | 42.682 (12.012) |
|  | 30-39 | 2.675 (0.957) | 2.005 (0.987) | 1.921 (0.981) | 2.385 (1.137) | 3.123 (0.874) | 3.151 (0.906) | 45.354 (10.261) |
|  | 40-49 | 2.724 (0.875) | 2.047 (0.924) | 1.909 (0.996) | 2.293 (1.099) | 2.988 (0.845) | 3.052 (0.904) | 46.063 (10.397) |
|  | 50-67 | 2.628 (0.902) | 1.889 (0.961) | 1.883 (0.939) | 2.011 (1.059) | 2.877 (0.866) | 2.900 (0.947) | 48.213 (10.240) |
| edu-  cation | high | 2.688 (0.859) | 1.970 (0.929) | 1.670 (0.870) | 2.276 (1.098) | 3.095 (0.822) | 3.132 (0.863) | 46.857 (10.285) |
|  | medium | 2.581 (0.919) | 1.879 (0.966) | 1.965 (0.982) | 2.265 (1.132) | 2.943 (0.865) | 2.991 (0.941) | 46.049 (10.906) |
|  | low | 2.576 (0.975) | 1.883 (1.041) | 2.146 (1.063) | 2.547 (1.169) | 2.985 (0.926) | 2.941 (1.021) | 44.294 (12.005) |

**Table A2.** Weighted correlations between PIPS in different life domains and MHRQOL

| variable | (1) | (2) | (3) | (4) | (5) | (6) | (7) | (8) |
| --- | --- | --- | --- | --- | --- | --- | --- | --- |
| education (1) | 1.00 | 0.05* | 0.04* | -0.16* | -0.04* | 0.07* | 0.08* | 0.06* |
| family (2) | 0.05* | 1.00 | 0.43* | 0.25* | 0.19* | 0.38* | 0.23* | -0.24* |
| partnership (3) | 0.04* | 0.43* | 1.00 | 0.24* | 0.13* | 0.24* | 0.14* | -0.18* |
| financial situation (4) | -0.16* | 0.25* | 0.24* | 1.00 | 0.24* | 0.22* | 0.12* | -0.19* |
| work/school (5) | -0.04* | 0.19* | 0.13* | 0.24* | 1.00 | 0.30* | 0.21* | -0.20* |
| social life (6) | 0.07* | 0.38* | 0.24* | 0.22* | 0.30* | 1.00 | 0.48* | -0.25* |
| leisure activity (7) | 0.08* | 0.23* | 0.14* | 0.12* | 0.21* | 0.48* | 1.00 | -0.18* |
| MHRQOL (8) | 0.06* | -0.24* | -0.18* | -0.19* | -0.20* | -0.25* | -0.18* | -1.00 |

*Note*. *significant at a 5% significance level, p- values were calculated using sample-weighted bootstrapping procedure

**Table A3** Adjusted generalized variance inflation factor for treatment, mediators, and covariates

| model | education | mediator | sex | age | migration background |
| --- | --- | --- | --- | --- | --- |
| Model 1 | 1.008 |  | 1.001 | 1.037 | 1.015 |
| Model 2a | 1.039 |  | 1.004 | 1.051 | 1.014 |
| Model 2b | 1.009 | 1.024 | 1.027 | 1.039 | 1.016 |
| Model 3a | 1.051 |  | 1.004 | 1.078 | 1.018 |
| Model 3b | 1.008 | 1.004 | 1.005 | 1.033 | 1.014 |
| Model 4a | 1.024 |  | 1.011 | 1.043 | 1.019 |
| Model 4b | 1.017 | 1.013 | 1.001 | 1.037 | 1.016 |
| Model 5a | 1.026 |  | 1.007 | 1.040 | 1.007 |
| Model 5b | 1.008 | 1.042 | 1.005 | 1.080 | 1.016 |
| Model 6a | 1.016 |  | 1.005 | 1.038 | 1.014 |
| Model 6b | 1.008 | 1.011 | 1.005 | 1.043 | 1.016 |
| Model 7a | 1.029 |  | 1.006 | 1.053 | 1.014 |
| Model 7b | 1.017 | 1.027 | 1.015 | 1.047 | 1.014 |

*Note.* Model 1: MHRQOL 🡨 education + covariates

Model 2a: PIPSfamily 🡨 education + covariates

Model 2b MHRQOL 🡨 education + PIPSfamily + covariates

Model 3a: PIPSpartnership 🡨 education + covariates

Model 3b MHRQOL 🡨 education + PIPSpartnership + covariates

Model 4a: PIPSfinancial situation 🡨 education + covariates

Model 4b MHRQOL 🡨 education + PIPSfinancial situation + covariates

Model 5a: PIPSwork/school 🡨 education + covariates

Model 5b MHRQOL 🡨 education + PIPSwork/school + covariates

Model 6a: PIPSsocial life 🡨 education + covariates

Model 6b MHRQOL 🡨 education + PIPSsocial life + covariates

Model 7a: PIPSleisure activity 🡨 education + covariates

Model 7b MHRQOL 🡨 education + PIPSleisure activity + covariates

**Table A4.** Results of the test for statistical significance of the treatment-mediator interaction

| PIPS life domain | p-value |
| --- | --- |
| family | 0.528 |
| partnership | 0.200 |
| financial situation | 0.352 |
| work/school | 0.996 |
| social life | 0.542 |
| leisure time | 0.832 |

*Note*. Results adjusted for sex, age, and migration background.

**Table A5.** Results of the mediation analysis (n = 5,855) of educational differences in MHRQOL due to PIPS in different life domains, including pre-pandemic MHRQOL as an exposure-induced mediator-outcome confounder

| PIPS life domain | effect type | beta | 95% CI | p-value |
| --- | --- | --- | --- | --- |
| family | ACME | **-0.525** | [-0.752; -0.060] | < 0.05 |
|  | ADE | **2.127** | [0.653; 3.260] | < 0.01 |
|  | Total effect | **1.602** | [0.206; 2.910] | < 0.05 |
| partnership | ACME | -0.239 | [-0.511; 0.100] | 0.250 |
|  | ADE | **1.811** | [0.432; 3.110] | < 0.05 |
|  | Total effect | **1.572** | [0.201; 2.940] | < 0.05 |
| financial situation | ACME | **1.184** | [0.856; 1.610] | < 0.001 |
|  | ADE | 0.425 | [-1.132; 1.670] | 0.696 |
|  | Total effect | **1.610** | [0.155; 2.880] | < 0.05 |
| work/school | ACME | 0.224 | [-0.277; 0.190] | 0.714 |
|  | ADE | **1.378** | [0.214; 2.900] | < 0.05 |
|  | Total effect | **1.602** | [0.155; 2.860] | < 0.05 |
| social life | ACME | **-0.885** | [-1.128; -0.500] | < 0.001 |
|  | ADE | **2.465** | [ 1.022; 3.560] | < 0.001 |
|  | Total effect | **1.580** | [0.169; 2.840] | < 0.05 |
| leisure time | ACME | **-0.616** | [-0.901; -0.340] | < 0.001 |
|  | ADE | **2.340** | [0.820; 3.450] | < 0.01 |
|  | Total effect | **1.724** | [0.154; 2.870] | < 0.05 |

*Note*. CI = confidence interval; ACME = average causal mediation effect; ADE = average direct effect. Significant coefficients (considering a .05 significance level) appear in bold. Results adjusted for sex, age, migration background and pre-pandemic MHRQOL.

**Table A6.** Comparison of the RKI-SOEP-2 sample (S_1_) and the final sample (S_2_)

| characteristics | | original sample (S_1_) | | | final sample (S_2_) | | | S_1_ vs. S_2_ |
| --- | --- | --- | --- | --- | --- | --- | --- | --- |
|  |  | mean | SD | N | mean | SD | N | ES^1^ or Pop diff^2^ |
| MHRQOL | 2021/2022 | 46.933 | 10.741 | 10,616 | 46.177 | 10.817 | 7,425 | -0.070^1^ |
| PIPS | family | 2.590 | 0.925 | 10,847 | 2.621 | 0.904 | 7,425 | 0.033^1^ |
|  | partnership | 1.843 | 0.967 | 10,771 | 1.914 | 0.961 | 7,425 | 0.074^1^ |
|  | finances | 1.783 | 0.934 | 10,739 | 1.883 | 0.962 | 7,425 | 0.107^1^ |
|  | work/school | 2.152 | 1.151 | 10,678 | 2.297 | 1.126 | 7,425 | 0.126^1^ |
|  | social life | 2.929 | 0.891 | 10,792 | 3.004 | 0.858 | 7,425 | 0.085^1^ |
|  | leisure time | 2.946 | 0.964 | 10,838 | 3.039 | 0.924 | 7,425 | 0.096^1^ |
| sex | male |  |  | 5,161 |  |  | 3,327 | -1,834^2^ |
|  | female |  |  | 6,001 |  |  | 4,098 | -1,903^2^ |
| age group | 18-29 |  |  | 1,319 |  |  | 1,060 | -259^2^ |
|  | 30-39 |  |  | 1,265 |  |  | 1,110 | -155^2^ |
|  | 40-49 |  |  | 1,827 |  |  | 1,643 | -184^2^ |
|  | 50-67 |  |  | 3,991 |  |  | 3,612 | -379^2^ |
|  | >67 |  |  | 2,760 |  |  | 0 | -2,760^2^ |
| migration background | none |  |  | 9,316 |  |  | 6,191 | -3,125^2^ |
|  | direct |  |  | 1,173 |  |  | 7,91 | -382^2^ |
|  | indirect |  |  | 604 |  |  | 443 | -161^2^ |
|  | NA |  |  | 69 |  |  | 0 | -69^2^ |
| education | high |  |  | 4,061 |  |  | 3,096 | -965^2^ |
|  | medium |  |  | 4,948 |  |  | 3,657 | -1,291^2^ |
|  | low |  |  | 967 |  |  | 672 | -295^2^ |
|  | NA |  |  | 1,186 |  |  | 0 | -1,186^2^ |

*Note.* S_1_ = original RKI-SOEP-2 sample (n = 11,162); S_2_ = final sample after age restriction and deletion of missing values (NA) (n = 7,425); SD = standard deviation; N = unweighted number of participants; ^1^ES = experimental selectivity; ^2^Pop diff = population difference; NA = missing values (not available).
